# Clinical practice: recognizing child sexual abuse—what makes it so difficult?

**DOI:** 10.1007/s00431-018-3193-z

**Published:** 2018-06-25

**Authors:** Thekla F. Vrolijk-Bosschaart, Sonja N. Brilleslijper-Kater, Marc A. Benninga, Ramón J. L. Lindauer, Arianne H. Teeuw

**Affiliations:** 1 Department of Social Pediatrics, Child Abuse and Neglect Team, Emma Children’s Hospital, Academic Medical Center Amsterdam, AMC, Meibergdreef 9 (h7-288), 1105 AZ Amsterdam, the Netherlands; 2 Department of Pediatric Gastroenterology, Emma Children’s Hospital, Academic Medical Center, Amsterdam, the Netherlands; 3 Department of Child and Adolescent Psychiatry, Academic Medical Center, Amsterdam, the Netherlands; 4 De Bascule, Academic center for child and adolescent psychiatry, Amsterdam, the Netherlands

**Keywords:** Child sexual abuse, Recognizing, Diagnostics

## Abstract

Recognizing child sexual abuse (CSA) in children is difficult, as there can be many hurdles in the assessment of alleged CSA. With this paper, we try to improve the recognition of CSA by discussing: (1) the difficulties regarding this matter and (2) the diagnostic evaluation of alleged CSA, combining both practical clinical recommendations based on recent research. Children are restrained to disclose CSA due to various reasons, such as fears, shame, and linguistic or verbal limitations. Associations between CSA and urogenital or gastrointestinal symptoms, internalizing and externalizing behavioral problems, post-traumatic stress symptoms, and atypical sexual behavior in children have been reported. However, these symptoms are non-specific for CSA. The majority of sexually abused children do not display signs of penetrative trauma at anogenital examination. Diagnosing a STI in a child can indicate CSA. However, other transmission routes (e.g., vertical transmission, auto-inoculation) need to be considered as well.

*Conclusion*: The assessment consists of medical interview and child interview (parents and child separate and together) with special attention to the child’s development and behavior (problems), psychosocial situation and physical complaints, the child’s mental health, and the child’s trauma history; anogenital examination should be done in all cases of alleged CSA. The examination should be documented by photo or video graphically. Recent research suggests that videography may be the preferred method, and testing on STIs. The assessment should be done multidisciplinary by experienced professionals. Health-care professionals who care for children need to know how child protective agencies and law enforcement are organized. In case there are concerns about a child’s safety, the appropriate authorities should be alarmed.

**Table:** 

**What is Known**: • *Sexual abuse in children often remains unrecognized in the majority of cases.* **What is New**: • *Research suggests that videographic documentation is preferred above photographic documentation for anogenital examination; observations of children’s behavioral reactions during examinations might be valuable in the evaluation of suspected sexual abuse; nucleic acid amplification testing can be used on vaginal swabs or urine samples for chlamydia and gonorrhea; the CRIES-13 and the CAPS-CA can be used to assess trauma-symptoms in children after sexual abuse.*

## Introduction

About 40 years ago, Kempe unveiled the problem of child sexual abuse (CSA) for pediatricians [29]. Although the problem might not be as hidden as in 1978, it remains a pediatric problem of high concern with many challenges concerning early recognition. The assessment of alleged CSA can be compared with putting together a puzzle, with various pieces representing for instance the medical and psychosocial history, physical examination, and laboratory findings.

In 1999, CSA was defined by the World Health Organizations as “Child sexual abuse is the involvement of a child in sexual activity that he or she does not fully comprehend, is unable to give informed consent to, or for which the child is not developmentally prepared and cannot give consent, or that violates the laws or social taboos of society. Child sexual abuse is evidenced by this activity between a child and an adult or another child who by age or development is in a relationship of responsibility, trust or power, the activity being intended to gratify or satisfy the needs of the other person. This may include but is not limited to: the inducement or coercion of a child to engage in any unlawful sexual activity; the exploitative use of a child in prostitution or other unlawful sexual practices; the exploitative use of children in pornographic performance and materials”. The exact prevalence of CSA remains unknown. It is estimated that the worldwide prevalence of CSA ranges from 3 to 31% [7, 43]. Girls appear to have a twofold higher risk of becoming CSA victims compared to boys [43]. Prevalence studies of CSA in young children (infants and preschoolers under the age of 6 years) are limited [7].

The impact of CSA later in life can be significant (varying from consequences for general health, gastrointestinal-, gynecologic- or reproductive health, pain, cardiopulmonary symptoms, to chronic pain obesity, and psychological symptoms and psychiatric disorders [24, 35]); therefore, it is important that CSA is recognized at an early stage in order to stop the abuse and offer adequate support. Unfortunately, many cases remain unrecognized. It is estimated by the Children’s Commissioner for England who investigated the CSA in 2017 that only one in eight victims of CSA come to the attention of statutory authorities. These data suggest that the scale of the problem is even larger than assumed based on the figures of authorities. Why is it so difficult for, among others, pediatricians to put the puzzle together? In short, we will discuss the most important difficulties as the regular absence of a disclosure, the relative value of the diverse and non-specific physical and psychosocial symptoms, and the relative value of the anogenital examination and STI tests.

Subsequently, we will discuss the diagnostic evaluation of alleged CSA. In the guidelines by Adams et al. (2015 and 2017), the focus is mainly on the interpretation of medical findings (e.g., results of physical examination and laboratory testing) [2, 3], which is also the main interest of the extensive reviews by the Royal College of Paediatrics and Child Health [38]. We found two papers by Kellog et al. (2005) and Jenny et al. (2013), in which the entire pediatric assessment is addressed including medical history, child interview, physical examination, laboratory testing, and the forensic evaluation [26, 27]. Both papers give clear clinical recommendations on how to evaluate CSA. In this present paper, we provide the readers with a combination of both practical clinical recommendations and indications how to interpret findings based on recent research.

## What makes it so difficult to recognize CSA in children?

The four factors that make it difficult for clinicians are the lack of disclosures, the diverse and non-specific physical and psychosocial symptoms, and the relative value of the anogenital examination and STI tests.

### Lack of disclosure

Although a child’s disclosure of CSA remains the most specific indicator of CSA [3], most children do not disclose spontaneously or only at a later stage, sometimes even adult age [17, 36]. Feelings of shame and guilt, fears for being blamed, or self-blame often withhold children from disclosing [17, 36]. In younger, preschool children, there are verbal limitations that might retain them from telling [8].

Considering the difficulties for children to disclose CSA, clinicians have to rely on other signs to recognize it. Suspicions of CSA may arise from the presence of physical and psychosocial symptoms in children. However, what is the scientific substantiation of physical and psychosocial symptoms in children victim of CSA?

### Non-specific symptoms

Traumatic experiences, such as CSA, may cause psychosocial symptoms in children, such as behavioral problems, post-traumatic stress symptoms, and depressive symptoms. This is associated with the effect of trauma on the developing brain in children and adolescents. Stressful experiences in childhood, such as CSA, may result in a dysregulated hypothalamic–pituitary–adrenal (HPA) axis (e.g., the stress pathway). And on its turn, a dysregulated HPA axis function has been linked to emotional psychopathology including anxiety, depression, and post-traumatic stress disorder (PTSD) [44].

Associations have been reported between CSA and behavioral problems, PTSD [25, 30, 40], increased risk of attempted suicide [18], self-injurious, and suicide-related behaviors in children [25, 30, 40]. Additionally, age-inappropriate sexual behavior is observed in about one third of children who have been sexually abused and is therefore believed to be an indication of possible CSA [6, 13, 16, 30]. However, age-inappropriate sexual behavior is also associated with many other traumatic experiences (e.g., physical abuse and other types of maltreatment, family violence, coercive parenting, child behavior, and modeling of sexual behavior) and thus non-specific for CSA [8, 9, 18, 28]. Age-inappropriate sexual knowledge is said to be a stronger indication for CSA [8, 48].

Regarding the physical consequences of CSA, many studies indicate a negative impact on physical health**.** Urogenital complaints (e.g., genital pain, dysuria and genital bleeding, and incontinence problems) [5, 10, 11] are reported more often in abused children. Studies on gastrointestinal complaints (abdominal pain, fecal incontinence, and constipation) and CSA give inconsistent data [5, 37, 45].

In short, a clear pattern is lacking for physical and psychosocial symptoms and none of the discussed symptoms are specific for CSA, other underlying causes than CSA are possible.

### Anogenital examination

Only few physical findings are highly suggestive of sexual abuse (even in the absence of a disclosure from the child, unless the child and/or caretaker provides a timely and plausible description of accidental anogenital injury, or past surgical interventions that are confirmed): acute laceration(s) or bruising of labia, penis, scrotum, or perineum; acute laceration of the posterior fourchette or vestibule, not involving the hymen; bruising, petechiae, or abrasions on the hymen; acute laceration of the hymen, of any depth, partial or complete; vaginal laceration; and perianal laceration with exposure of tissues below the dermis [3, 38].

There is no consensus on the weight to be given, with respect to sexual abuse, for complete anal dilatation (in the absence of other predisposing factors such as constipation, encopresis, sedation, anesthesia, and neuromuscular condition), and hymenal notches or clefts at or below the three o’clock or nine o’clock location [3].

We need to emphasize that CSA-specific findings are only found in a minority of CSA victims (4–5%) when examined over 72 h after the last abuse [1, 2, 21, 22, 38, 49], due to the rapid healing of mucous tissue. Accordingly, in 95%, there are no physical findings at anogenital examination in children examined 48 h or more after the abuse [1, 2].

### Sexually transmitted infections

The prevalence of sexually transmitted infections (STIs) among children is low [3] and in most cases of alleged CSA STI-tests are negative. Diagnosing a sexually transmitted infection (STI) in a child does not necessarily mean the child has been sexually abused. The entire context needs to be taken into account. Some infections are not related to sexual contact, such as vaginitis caused by fungal infections, bacterial infections transmitted by non-sexual means (Streptococcus species, Staphylococcus sp., *E. coli*, Shigella, or other gram-negative organisms), or genital ulcers caused by viral infections such as Epstein Barr virus or other respiratory viruses [3].

Multiple infections can both be by non-sexual and sexual transmission: Molluscum contagiosum in the genital or anal area, Condyloma acuminatum (HPV) in the genital or anal area, and herpes simplex type 1 or 2 infections in the oral, genital, or anal area [3, 38]. Additional information is required to interpret these infections to clarify the likelihood of sexual transmission.

For gonorrhea, chlamydia, syphilis, Trichomonas vaginalis, and HIV infections, sexual contact is the most likely transmission route if the infection is confirmed by appropriate testing, and perinatal transmission has been ruled out (and for HIV if also transmission by blood or contaminated needles has been ruled out) [3, 38]. However, in adolescents, additionally consensual sexual contact with peers needs to be ruled out before concluding on CSA. Another complicating factor is the fact that there is a high false positive rate in populations with low prevalence of STIs [3]. This should always be considered and therefore confirmation tests are required.

### Recommendations concerning the evaluation of alleged CSA

Generally, children do not disclose CSA and so far, there has not been a recognizable pattern of both physical and psychosocial symptoms in children after CSA. Additionally, anogenital abnormalities specific for CSA are rarely found. Also diagnosing an STI does not necessarily mean a child has been sexually abuse. Only a pregnancy or semen identified in forensic specimens taken directly from a child’s body are diagnostic for sexual contact (but not necessarily CSA) [3]. When CSA is suspected, the physician needs to coordinate the care with other professionals to provide the most complete and accurate assessment possible.

### The first response

Referring every child with psychological or unexplained physical symptoms for CSA evaluation, because CSA is in the differential diagnosis, is impractical. Clinicians who care for these children need to be aware of the diverse and non-specific physical and psychosocial symptoms which can be displayed in CSA-victims and should be alerted by other signs or symptoms matching with CSA. If other signs and symptoms are noted, that are consistent with CSA, direct referral for further evaluation is required. Examples are a disclosure of CSA, anogenital injury not explained by an accident, pregnancy without a history of consensual sexual contact with a peer, and/or a STI not explained by non-sexual transmission. Other examples where referral is in its place are as follows: children diagnosed with a PTSD, without explanatory event; children showing atypical behavioral problems, without explanation, for example attention deficit hyperactivity disorder, or atypical sexual behavior; or when children show age-inappropriate sexual knowledge.

It is important that alleged CSA in children is evaluated systematically, including a medical and psychosocial history, physical examination (including anogenital examination), child interview (including traumatic history) and screening for (other) STIs. The assessment should be done multidisciplinary (including at least medical and psychological expertise) by experienced clinicians with knowledge of the facts on issues related to CSA and other types of maltreatment, age-appropriate child (sexual) development, physical symptoms, and (normal) anogenital anatomy.

Jenny et al. underline the importance to always address the following five issues whenever the issue of alleged CSA arises: (1) the child’s safety, (2) reporting to protective services, (3) the child’s mental health, (4) the need for physical examination, and (5) the need for forensic evaluation [26]. We add a sixth issue, which should be considered separately, namely, the need for testing on STIs.

The child’s safety: whenever the question of alleged CSA arises, there are worries about a child’s safety the appropriate authorities should be alarmed. In the Netherlands, health-care professionals have the possibility to anonymously discuss a case and evaluated whether to be concerned for the child’s safety with a professional from the child abuse investigating agencies. How child protective agencies and law enforcement are organized differs per country and state. Nevertheless, we would advise to always discuss the issue of a child’s safety with at least another colleague.

When and where to report a case of (suspected) CSA is dependent on the country and state one works in. Therefore, every child health-care professional needs to be advised of the child abuse reporting laws and how child protective agencies are organized in their country or state. When pediatricians have a reasonable suspicion that the child was abused, this should be reported. Subsequently, it is up to the child protective services agency to conduct a thorough investigation to determine whether abuse has occurred [26].

The need for forensic evaluation depends on the time between the last event and consultation. For a long time, 72 h between the last event and consultation was applied as the time limit to decide whether forensic evaluation (such as DNA-sampling for legal evidence) was possible. Today, with the improvement of technology, the time limit is expended up to 7 days [38]. In acute cases of alleged CSA, the child needs to be seen as soon as possible if collecting forensic evidence might be possible. Acute presentations of alleged CSA are relatively rare in children, mainly due to the absence of children’s disclosures. The remaining three issues will be discussed in the following paragraphs.

### Medical history—talking with parents, conflict divorces

Talk with the parents separately from the child to prevent that the child is influenced by what he/she hears from her parents [26]. In the conversation with parents, it is possible to address concerns on (normal) sexual behavior, parental relationship issues, and parent’s own history of abuse which might influence their concerns. It is essential to document carefully and in details the parent’s concerns and the source of information [26].

Taking a full medical history including detailed description of a child’s development and behavior (problems), psychosocial situation, and physical complaints is part of the work-up for alleged CSA. Knowledge on age-appropriate child (sexual) development and psychopathology in children is required [8]. Clinicians need to be alert for (sexual) behavioral problems, regression in development, and unexplained physical complaints (such as anogenital and gastrointestinal symptoms). Nevertheless, there is no specific pattern of symptoms, neither psychosocial nor physical, and the absence of symptoms never excludes CSA.

Often concerns for CSA arise based on sexual behaviors displayed in children. What is considered “typical” sexual behavior depends on a child’s age and developmental stage. Two-year-old children display relatively overt sexual behavior and it increases up to age five than do 10- to 12-year-olds, and sexual behavior increases up to age five [8, 15]. Preschool children are unaware of social norms and do not have feelings of shame. As children become older, they learn which behavior is and which behavior is not appropriate in public. This results in a decrease of observed sexual behavior in children [8]. In general, “typical” childhood sexual play happens between children of approximately the same age and developmental level, spontaneously, intermittently, with mutual consent, and does not cause any distress or pain [9]. In most situations, even though they might concern parents, the sexual behaviors in young children do not require child protective services intervention. In these cases, the pediatrician may provide education, guidance in supervision, and monitoring of the behavior [26, 28]. More comprehensive assessment is warranted (assessment of all family and environmental factors and investigation by a child protective services) in children aged 2–6 years in whom sexual behaviors involve children who are 4 or more years apart; a variety of sexual behaviors are displayed on a daily basis; sexual behavior results in emotional distress or physical pain, is associated with other physically aggressive behaviors, or involves coercion; or the sexual behaviors are persistent and child becomes angry if distracted [28].

It is always a possibility that concerns for CSA arise due to interparental conflicts or relationship issues. Whether or not this is a big issue in cases of alleged CSA is difficult to say as there is not much scientific research available on this topic. However, in a study on the clinical profiles of children assessed for alleged child sexual abuse, in one third of the children, it was a case of conflict divorce. Nevertheless, all concerns for alleged CSA should be evaluated objectively, thoughtfully, and with an open mind [26].

### Child interview

The details of the disclosure of abuse from the child is the most important part of an evaluation, whether or not a physical or laboratory finding is present [3].

Interviewing children on traumatic experiences needs to be done carefully by skilled professionals trained to interview children about possible abuse [8], separate from the child’s parents [26]. How children are interviewed influences the positive and negative predictive value of abuse disclosure [33]. In cases where the police is involved (or likely to become involved), it is advised to deliberate with the police on who will interview the child.

The interviewer needs to approach the child at his own level in terms of development, readiness to disclose, culture, and language [14]. Misleading questions need to be avoided as they generally increase false positive rates, especially in younger/preschool children. Free recall questions are preferred above direct questions with the use of tools such as anatomical dolls. When open-ended questions are used, children’s free recall reports of genital touch are much more probative than “yes” responses to closed-ended questions [33]. Other recommendations for interviewing children about alleged abuse are as follows: giving clear instructions to the child about how to respond (the child may say “I don’t know, I don’t understand” or correct the interviewer when needed); making a promise to tell the truth (increases honesty without increasing errors [33]); use of secret instructions (e.g., distinguishing between good/bad secrets and encouraging disclosure of bad secrets); which can increase disclosure early in free recall [4]); and using “How” questions (e.g., How did you feel?) (likely to elicit evaluative information [34]). If a child starts talking only yes/no, questions like “tell me more” and “what happened next” should be used. A child should never be urged or coerced to talk about abuse [26].

In case the child gives a disclosure of CSA during the interview, the interviewer should respond that it is OK to talk about abuse with an adult [26]. It can also be helpful if the child’s parents tell the child that it is allowed to talk with the clinician on forehand. The disclosure should be carefully recorded in the child’s record, using the child’s own language, quotation marks, and preferably together with the question or the note that it was a spontaneous disclosure [26]. If possible, video recordings of the interview can be helpful.

Besides obtaining abuse-specific information, the child interview should be used to talk about other traumatic experiences and to assess a child’s mental health. Experiencing abuse may lead to depressive symptoms or post-traumatic stress in a child. Additionally, also the disclosure itself might cause serious distress in a child. The Children’s Revised Impact of Event Scale (CRIES-13) can be used by health-care professionals, also if not specialized in mental health [46, 47]. The CRIES-13 is a validated instrument for the early detecting of traumatic complaints (parent and child version) [46, 47]. For health-care professionals specialized in mental health, the Clinician Administered PTSD Scale for Children and Adolescents (CAPS-CA) can be used [12]. This is a structured clinical interview and considered as the gold standard for the assessment of PTSD [12]. Emergent and appropriate mental health care should be offered in case of PTSD or depressive symptoms [26].

### Physical examinations

As stated above, only few physical findings are highly suggestive of sexual abuse [3]. The absence of physical findings never excludes CSA [1, 2, 21, 22, 38, 49]. In any case of alleged CSA, a thorough anogenital examination should be performed, especially if the child reports anogenital complaints [26]. Physically examining children for alleged CSA requires training and special skills. As children who have experienced one type of abuse also are at risk for other types of abuse or neglect, the anogenital examination should always be joined with a general physical exam [26].

The appearance of the hymenal rim may change with examination position or technique; children should always be examined in both supine and the knee chest position; the use of intravaginal instruments (such as a speculum) is not necessary in most cases and contra-indicated in pre-pubertal girls [3, 26]. If intravaginal trauma is suspected, vaginoscopy should only be performed under anesthesia [3, 26].

As it is difficult to interpreting anogenital findings, the anogenital examinations should always be documented photo- or videographically. This allows clinicians to consult (forensic) experts without burdening a child with additional examinations. Video recordings can document the examination in a dynamic state. The use of video recordings, compared to still photos, shows significantly greater agreement between clinician on the diagnosis of a hymenal transection. These results suggest that videography may be a preferred method for documenting findings in cases of alleged CSA [3, 31].

When injuries are found in the anogenital area, it is important that accidental injuries, inflicted injuries, illnesses (such as lichen sclerosis or urethral prolapse), normal anatomical variances, and CSA are included in the differential diagnosis. Knowledge on illnesses like lichen sclerosis, urethral prolapse, and the wide variation in normal anatomy is crucial.

The majority of non-abused children perceives the anogenital examination as neutral or expresses some minor symptoms of distress [19]. Two other studies found that the anogenital examination was not perceived negative by the examined children [32, 42]. Children with anxious feelings prior to the examination seem to experience more anxious feelings during the examination [39]. Careful explanation of the examination can decrease symptoms of anxiety during the examination [39, 50].

In children examined because of alleged CSA, the majority does not report clinically significant levels of anxiety or emotional distress either before or after the medical evaluation [23, 41]. Nonetheless, a noteworthy amount of children report moderate to severe levels of anxiety, suggesting that interventions to reduce anxiety seem warranted [41]. Also, more emotional distress and anxiety can be found in children who possibly experienced more extensive and aggravated events during the sexual abuse or give history of more invasive forms of sexual abuse [20, 23].

### Laboratory testing

It is not necessary to test on STIs in every case of alleged CSA. The risk of contaminating a STI depends on various factors such as the nature of the abuse and the prevalence of STIs in the population. Further, the profile of the possible perpetrator and the presence of anogenital complaints or injuries should be considered when deciding on whether or not to test for STIs in children with alleged CSA [38]. The timing is dependent on the varying incubation times. STIs can be asymptomatic; thus, the absence of symptoms should not withhold from testing. Indications for testing on STIs are the following [3, 26]:

1. Child has experienced penetration of the genitalia or anus.
2. Child has been abused by a stranger.
3. Child has been abused by a perpetrator known to be infected with an STI or is at high risk for being infected (intravenous drug users, men who have sex with men, or people with multiple sexual encounters).
4. Child has a sibling or other relatives in the household with an STI.
5. Child lives in an area with a high rate of STI in the community.
6. Child has signs or symptoms of an STI.
7. Child has already been diagnosed with one STI.

For chlamydia and gonorrhea, nucleic acid amplification testing (NAAT) on vaginal swabs or urine samples is preferred over vaginal cultures [3]. In prepubertal children confirmatory testing with a second, alternate target NAAT should be considered, which is not necessary for adolescents [3]. For Trichomonas, culture is the most sensitive test that is readily available [3]. Which sides to test (e.g., vaginal, oral, and/or anal) should not depend on patient symptoms or reported type of sexual contact as this might result in missed gonorrhea or chlamydia infections [3].

When there is a gonorrhea, chlamydia, syphilis, Trichomonas vaginalis, or HIV infections, and sexual contact is the most likely transmission route (and in adolescents consensual sexual contact has been ruled out) [3, 38], CSA is likely and clinicians should report to child protective services agency to conduct a thorough investigation to determine whether abuse has occurred [26].

In case of positive tests for Molluscum contagiosum, Condyloma acuminatum (HPV), and herpes simplex type 1 or 2 infections [3, 38], additional information is required to interpret these infections to clarify the likelihood of sexual transmission. We advise clinicians to seek expert opinion in these cases.

## Conclusion

Although it has already been 40 years ago since Kempe unveiled the problem of CSA, it remains difficult for clinicians to recognize CSA. The lack of disclosures, a clear symptom pattern and often absence of specific indications for CSA make it more difficult to “diagnose” CSA. Adequate assessment of alleged CSA should be done multidisciplinary by experienced professionals and consists of the following:

1. Health-care professionals who care for children need to be aware on how child protective agencies and law enforcement are organized in their country and in case there are concerns about a child’s safety the appropriate authorities should be alarmed.
2. A thorough medical interview and child interview (parents and child separate) with special attention to a child’s development and behavior (problems), psychosocial situation and physical complaints, and the child’s trauma history and mental health.
3. Anogenital examination should be done in all cases of alleged CSA. The examination should be documented photo- or videographically. Recent research suggests that videography may be the preferred method.
4. NAAT on vaginal swabs or urine samples for chlamydia and gonorrhea, and vaginal cultures for Trichomonas are the preferred STI tests. Which sides to test (e.g., vaginal, oral, and/or anal) should not depend on patient symptoms or reported type of sexual contact.

## Abbreviations

CSA: Child sexual abuse
HPA: Hypothalamic–pituitary–adrenal
PTSD: Post-traumatic stress disorder
STI: Sexually transmitted infection
